# Improvements in Resistive and Capacitive Switching Behaviors in Ga_2_O_3_ Memristors via High-Temperature Annealing Process

**DOI:** 10.3390/ma17112727

**Published:** 2024-06-04

**Authors:** Hye Jin Lee, Jeong-Hyeon Kim, Hee-Jin Kim, Sung-Nam Lee

**Affiliations:** 1 Department of IT & Semiconductor Convergence Engineering, Tech University of Korea, Siheung 15073, Republic of Korea; 2 Department of Nano & Semiconductor Engineering, Tech University of Korea, Siheung 15073, Republic of Korea

**Keywords:** Ga_2_O_3_, memristor, capacitance, annealing, RF sputter

## Abstract

This study investigates the effect of a high-temperature annealing process on the characteristics and performance of a memristor based on a Ag/Ga_2_O_3_/Pt structure. Through X-ray diffraction analysis, successful phase conversion from amorphous Ga_2_O_3_ to β-Ga_2_O_3_ is confirmed, attributed to an increase in grain size and recrystallization induced by annealing. X-ray photoelectron spectroscopy analysis revealed a higher oxygen vacancy in annealed Ga_2_O_3_ thin films, which is crucial for conductive filament formation and charge transport in memristors. Films with abundant oxygen vacancies exhibit decreased set voltages and increased capacitance in a low-resistive state, enabling easy capacitance control depending on channel presence. In addition, an excellent memory device with a high on/off ratio can be implemented due to the reduction of leakage current due to recrystallization. Therefore, it is possible to manufacture a thin film suitable for a memristor by increasing the oxygen vacancy in the Ga_2_O_3_ film while improving the overall crystallinity through the annealing process. This study highlights the significance of annealing in modulating capacitance and high-resistive/low-resistive state properties of Ga_2_O_3_ memristors, contributing to optimizing device design and performance. This study underscores the significance of high-temperature annealing in improving the channel-switching characteristics of Ga_2_O_3_-based memristors, which is crucial for the development of low-power, high-efficiency memory device.

## 1. Introduction

Ga_2_O_3_ is highly valued in electronic and optical devices due to its properties, such as a wide band gap of 4.9 eV, excellent physicochemical properties, and thermal stability [1,2]. In general, Ga_2_O_3_ has five different atomic structures corresponding to α, β, γ, δ and ε, of which the beta phase is known to be the most stable [3]. Due to the stability of β-Ga_2_O_3_, various devices, such as capacitors, photodetectors, gas sensors, and resistive memory, are being studied and developed through various processes using the most stable structure of β-Ga_2_O_3_ [4,5,6,7,8]. With the recent increase in storage capacity, there has been a growing interest in capacitive and resistive memory for data and energy storage applications. Ga_2_O_3_ stands out as a promising material for such storage because it has a wide band gap, making it suitable as an excellent electrical insulator. Thus, its stability as an insulator suggests its suitability as a dielectric material for capacitors. Additionally, its diverse growth methods offer the advantage of ease of growth, through various techniques. By controlling growth conditions to adjust oxygen vacancies, Ga_2_O_3_ can also be used as a suitable material for resistive memory applications. In particular, a resistive random-access memory (RRAM) device based on Ga_2_O_3_ can operate based on resistive switching, enabling the device to freely transition between a high-resistance state (HRS) and a low-resistance state (LRS) [8,9]. RRAM consists of a simple structure, known as a metal-Ga_2_O_3_-metal structure, which is similar to that of a capacitor. Based on this, a memory capacitor leveraging resistance change was proposed [10]. Oxygen vacancies, an intrinsic defect in these Ga_2_O_3_-based resistance-dependent memories and capacitors, greatly affect the formation of filament channels, which is an important component of memristor and memcapacitor behaviors, and therefore, the behavior and performance of these devices [11,12]. The research groups are actively studying various aspects of Ga_2_O_3_ memristor and memcapacitor technology, including analyzing Ga_2_O_3_ properties, such as electrical conductivity, carrier concentration, and mobility [13,14,15,16]. To date, most research has focused on understanding the underlying mechanisms of resistive switching and improving device performance, durability, and scalability [17,18,19]. To improve these properties, studies are also underway to optimize the quality of insulator thin films using various vacuum deposition methods, such as chemical vapor deposition (CVD), atomic layer deposition, and sputtering [17,18,19,20,21]. Although sputtering does not guarantee the same film quality as ALD and CVD, it can still be a suitable deposition technique for the fabrication of electronic devices that do not require the excellent charge transport properties provided by epitaxial substrates [20]. In addition, an RF sputtering system precisely controls deposition parameters, such as sputtering power, Ar/O_2_ gas pressure, and substrate temperature, to enable reproducible growth of Ga_2_O_3_ films with desired properties. However, it is noted that the quality of the thin films may be relatively lower compared to those grown by CVD [21]. Therefore, oxide semiconductor thin films grown by RF sputtering can be easily applied to devices such as memristors because they easily form conductive filaments, due to intrinsic defects. However, an excess of crystal defects can pose challenges for device properties and reliability. Therefore, strategies such as growth pressure control or the adoption of laminated structure growth have been reported to improve the quality of thin films grown by RF sputtering [22,23]. In addition, the most effective way to improve the film quality is known to be thermal treatment [24]. The effects of recrystallization and defect control resulting from thermal treatment are expected to control the characteristics of high-performance memristors and memcapacitors, offering a straightforward and rapid method of improving thin film quality [24,25].

In the Ga_2_O_3_-based memristor, the quality of Ga_2_O_3_ thin film and the presence of oxygen vacancies significantly impact Ga_2_O_3_ memristor behavior [26], with recrystallization from amorphous to crystalline states leading to reduced leakage current and an increased on/off ratio, making it suitable for high-performance memory devices. In addition, oxide semiconductors with a wide bandgap, such as Ga_2_O_3_, can develop devices suitable for high-performance capacitors because recrystallization removes deep levels caused by crystal defects, resulting in lower leakage currents and higher-quality insulation properties [27,28]. Additionally, high-temperature annealing enhances the crystalline quality of Ga_2_O_3_ by reducing defects, and crucially affects the formation of oxygen vacancies. These vacancies are essential for creating and controlling conductive filaments necessary for resistive switching [29]. Unlike other semiconductor devices, Ga_2_O_3_-based memristors can switch between high-resistivity and low-resistivity states after annealing, although the additional oxygen vacancies created during annealing improve the overall crystallinity of the thin film. This dual role of annealing to improve crystal quality and optimize oxygen vacancies can be an important process in sputter-grown Ga_2_O_3_. Therefore, optimization of the oxygen vacancy concentration in Ga_2_O_3_ thin film can affect the performance of the capacitor and memristor, as well as channel formation and dissolution, which affect the resistance of the memristor [30,31]. The formation of conductive channels by controlling oxygen vacancies, which can affect resistance and capacitance, will be able to be controlled based on crystallization through thermal treatment of Ga_2_O_3_ thin films, which is crucial for the realization of highly efficient devices produced with Ga_2_O_3_-based memristors and memcapacitors. In this study, Ga_2_O_3_ thin films grown by RF sputter were annealed at various temperatures to control the crystal defect in the Ga_2_O_3_ thin films. The relationship between the resistive switch behavior and capacitance along the conductive filament channel of the metal/Ga_2_O_3_/metal structure was then systematically studied.

## 2. Materials and Methods

Prior to depositing Ga_2_O_3_ using RF sputtering, the c-plane sapphire substrate is cleaned using the RCA cleaning. This process involves ultrasonically treating substrates with acetone for 10 min, followed by isopropyl alcohol (IPA) for 10 min, and then deionized water for 10 min to remove organic impurities from the surface. Subsequently, the 50 nm thick bottom electrode of Pt is deposited using RF sputtering, followed by deposition of a 100 nm thick Ga_2_O_3_ thin film using RF sputtering with a high-purity Ga_2_O_3_ target. Before deposition, the base pressure of the chamber is pumped down to 8.7 × 10^−7^ Torr, with a working pressure of 3 × 10^−3^ Torr, RF power set at 100 W, and growth temperature maintained at room temperature. Following the growth of the Ga_2_O_3_ thin film, furnace annealing is performed at 400 °C, 600 °C, and 800 °C for 3 min in an air atmosphere. Subsequent to thermal treatment on the Ga_2_O_3_ thin film, a 50 nm thick Ag electrode is deposited as a top electrode using a thermal evaporator.

The surface structure of the Ga_2_O_3_ film grown by RF sputtering was characterized using AFM (NanoFocus, my-Scope plus, Seoul, Republic of Korea). High-resolution X-ray diffraction (HR-XRD) ω/2θ scan (PANalytical, X’Pert Pro MRD, Tokyo, Japan) was conducted to investigate the crystalline structures of the Ga_2_O_3_ film on the bottom metal. X-ray photoelectron spectroscopy (XPS) spectra obtained from the ThermoFisher Scientific (Waltham, MA, USA) NEXSA system were carefully analyzed to detect the presence of oxygen vacancies and to examine the chemical environment of the Ga_2_O_3_ elements on the surface of the films. The optical band gap of the Ga_2_O_3_ thin film was determined by measuring its optical absorbance in the UV-visible range (ThermoFisher Scientific, Evolution 300) of 200 nm to 800 nm. Electrical characterization, including leakage current and memristor behavior, was carried out using a Keithley 2614B source meter. The capacitance of the devices was characterized using a 4284A LCR meter, with frequencies ranging from 500 Hz to 1.0 MHz. Through these experimental techniques and characterization methods, we investigated the relationship between the characteristics of the LRS and high-resistance state (HRS) and the capacitance of the Ga_2_O_3_ memristors. Moreover, we analyzed the effect of furnace annealing temperature on the electrical behavior and performance of Ag/Ga_2_O_3_/Pt memristors.

## 3. Results and Discussion

**Figure 1:**
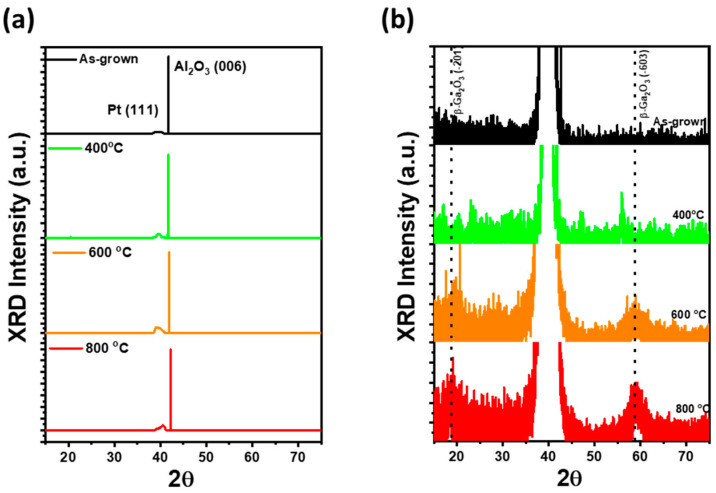
(**a**) HR-XRD ω/2θ broad scan spectra ranging from 15° to 75° for as-grown, 400 °C, 600 °C, and 800 °C annealed-Ga_2_O_3_/Pt/sapphire, and (**b**) measurement of ω/2θ spectra focused on the low-XRD-intensity region to identify weak β-Ga_2_O_3_ (−201) and (−603) peaks.

Figure 1a,b shows the results of HR-XRD ω/2θ scans of Ga_2_O_3_ thin films grown on Pt/sapphire templates at room temperature and after thermal treatment at 800 °C to investigate the crystallinity of the film, respectively. As shown in Figure 1a, Ga_2_O_3_/Pt/sapphire without thermal treatment, despite the presence of Ga_2_O_3_ thin film, no peaks related to Ga_2_O_3_ were observed, only the (111) Pt peak related to the bottom electrode and the (006) Al_2_O_3_ peak related to the sapphire substrate. It is clearly evident that the Ga_2_O_3_ thin film grown by sputtering was formed from an amorphous Ga_2_O_3_ thin film that does not exhibit good crystallinity with a single-crystal orientation due to low growth temperature and plasma damage [32]. For Ga_2_O_3_/Pt/sapphire annealed at temperatures above 400 °C, only the (111) Pt peak and (006) Al_2_O_3_ peak were observed in the broad XRD scan with high intensity, as depicted in Figure 1a. However, upon closer examination, in Figure 1b, where the XRD analysis was focused on a lower intensity range, peaks corresponding to (−201) and (−603) planes associated with β-Ga_2_O_3_ emerged around 18.4° and 59°, respectively. This indicates that the thermal treatment had preferentially oriented the amorphous Ga_2_O_3_ thin film toward the β-Ga_2_O_3_ plane, resulting in a significant improvement in crystallinity [33,34]. In particular, the XRD analysis in Figure 1b reveals the evolution of the β-Ga_2_O_3_ phase in the annealed Ga_2_O_3_ thin films. At 400 °C, very faint (−201) and (−603) β-Ga_2_O_3_ peaks were detected around 18.4° and 57°, respectively, suggesting that the β-Ga_2_O_3_ phase was first formed. Subsequently, as the annealing temperature was increased to 600 °C, the β-Ga_2_O_3_ phase associated with the (−201) and (−603) planes became more pronounced, with broader peaks near 18.4° and spanning 56° to 61°. Finally, at 800 °C, the intensity of the XRD peaks increased notably, accompanied by a reduction in full width at half maximum, indicative of further crystallization. These observations indicate a progressive enhancement in the formation and crystallinity of the β-Ga_2_O_3_ phase with increasing annealing temperature.

**Figure 2:**
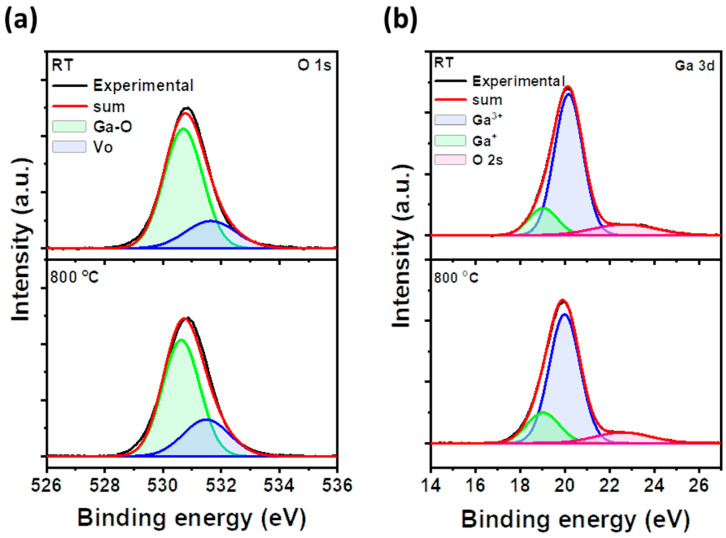
XPS (**a**) O1s and (**b**) Ga3d peaks of as-grown and 800 °C-annealed Ga_2_O_3_ thin films. The O1s were fitted with Ga-O and V_o_ peaks, and the Ga3d peaks were fitted with Ga^3+^, Ga^+^, and O2s. The black line represents the actual measurement. The green, blue, and pink lines correspond to the spectra fitted to the items shown in the legend of each figure.

XPS analysis was conducted on as-grown Ga_2_O_3_/Pt/sapphire and Ga_2_O_3_/Pt/sapphire annealed at 800 °C to characterize the defects of Ga and O atoms. Figure 2a,b shows the O1s and Ga3d peaks of as-grown and 800 °C-annealed Ga_2_O_3_ thin films, respectively. As shown in Figure 2a, the O1s spectrum of Ga_2_O_3_ demonstrated a satisfactory fitting with two peaks: Ga-O and oxygen vacancies. The Ga-O, located at a binding energy of 530.0 eV, corresponded to the O^2−^ ions surrounded by Ga atoms. Conversely, the Vo peak, positioned at a binding energy of 531.6 eV, was attributed to the presence of oxygen vacancies [35]. Upon thermal treatment at 800 °C, the ratio of Vo peak area (Vo/[Ga-O + Vo]) to the total area of the O1s peak increased from 23% to 28% for the as-grown Ga_2_O_3_ thin film. This increase was attributed to the volatilization of oxygen atoms with the Ga_2_O_3_ thin film during high-temperature thermal treatment, leading to an augmentation of oxygen vacancies [36]. Figure 2b shows the Ga3d peak of both as-grown and 800 °C-annealed Ga_2_O_3_ thin films. The Ga3d peak can be characterized by a Ga^3+^ peak at 20.0 eV and a Ga^1+^ peak at 18.6 eV. The Ga^3+^ peak corresponds to Ga within Ga_2_O_3_, while the Ga^1+^ peak represents Ga within the Ga_2_O phase [37]. When comparing the Ga^3+^ and Ga^+^ peaks of the as-grown and 800 °C-annealed Ga_2_O_3_ thin films, the Ga^3+^ratio (Ga^3+^/[Ga^3+^ + Ga^+^]) in the as-grown Ga_2_O_3_ showed 83.7%, which decreased to 80.0% after the 800 °C annealing process. Conversely, the Ga^+^ peak ratio (Ga^+^/[Ga^3+^ + Ga^+^]) in the as-grown Ga_2_O_3_ thin film was 16.3%, which increased to 20.0% following the 800 °C-annealing process. This indicates that the high-temperature annealing process at 800 °C caused the volatilization of oxygen from the Ga_2_O_3_ thin film, resulting in a reduction of the Ga_2_O_3_ phase and the formation of some Ga_2_O. This finding aligns with the O1s peak analysis of both the as-grown and 800 °C-annealed Ga_2_O_3_ thin film. Moreover, there was no significant formation of trace oxygen vacancies and, thus, no substantial formation of the Ga_2_O phase. Considering the HR-XRD results in Figure 1, this indicates that despite the formation of oxygen vacancies and Ga_2_O phase, the high-temperature annealing process can produce β-Ga_2_O_3_ thin films with relatively good crystal quality from initially amorphous Ga_2_O_3_ thin films.

**Figure 3:**
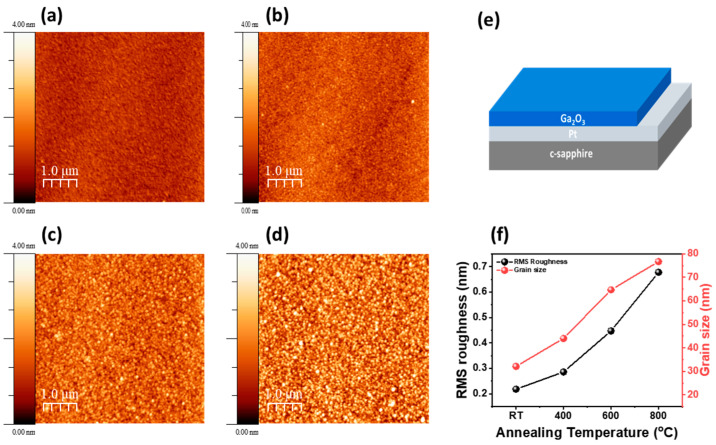
Surface morphologies of (**a**) as-grown, (**b**) 400 °C-, (**c**) 600 °C-, and (**d**) 800 °C-annealed Ga_2_O_3_ thin film, measured using AFM. (**e**) Schematic of Ga_2_O_3_/Pt structure grown on sapphire substrate. (**f**) Surface RMS roughness of Ga_2_O_3_ films as a function of annealing temperature.

As shown in Figure 3, the surface morphology of Ga_2_O_3_ thin films deposited on the (0001) sapphire was analyzed using AFM. With annealing temperature increasing from 25 °C to 800 °C, the average size of the surface grains of Ga_2_O_3_ thin films increased from 32.1 nm to 76.7 nm. In addition, as depicted in Figure 3e, the root-mean-square (RMS) roughness of the surface increased from 0.22 nm to 0.68 nm as the annealing temperature increased from 25 °C to 800 °C, respectively. The increase in the size of surface grains and surface roughness of Ga_2_O_3_ thin films during this high-temperature annealing process can be attributed to the vibration and movement of atoms induced by the high-temperature recrystallization of the Ga_2_O_3_ structure [38]. The post-nucleation growth of the island structure was attributed to the low surface mobility of atoms adsorbed during the sputtering process. Subsequent recrystallization through the Oswald-ripening effect, like grain growth during the thermal treatment process, is believed to crystallize the initial amorphous Ga_2_O_3_ into β-Ga_2_O_3_. This transformation is expected to improve the structural quality and electrical properties of the thin film [39].

**Figure 4:**
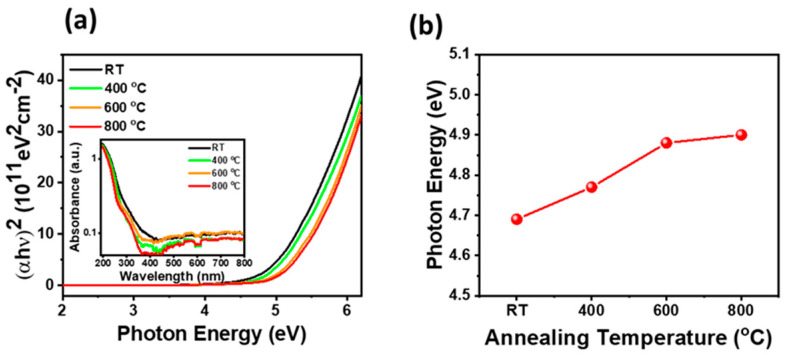
(**a**) Plot of (αhν)^2^ against photon energy for the Ga_2_O_3_ films with different annealing temperatures. The inset shows the absorbance spectra of Ga_2_O_3_ thin films annealed at different temperatures. (**b**) Energy bandgap of Ga_2_O_3_ films as a function of the annealing temperature ranging from 25 °C to 800 °C.

Figure 4a shows the Tauc plot of the Ga_2_O_3_ thin films with different thermal treatments as a function of photon energy. The energy bandgap of Ga_2_O_3_ films under different thermal treatments can be determined using the equation α*h*ν = B(*h*ν − E_g_)^1/2^, where α is the absorption coefficient, *h* is the Planck constant (4.135 ×10^−15^ eVs), ν denotes the frequency (s^−1^), B is a constant, and E_g_ represents the energy band gap (eV) [40]. As the annealing temperature increased, it is apparent that the slopes for high energies above 5.0 eV were nearly identical but shifted towards higher energies. This implies that the optical bandgap of the Ga_2_O_3_ thin film expands with an increasing in the annealing temperature due to the improvement of crystallinity. More specifically, the optical bandgap energy of the Ga_2_O_3_ thin film was calculated by extrapolating the linear portion of the curve to the energy axis, as shown in Figure 4b. As the annealing temperature increased from 25 °C to 800 °C, the optical bandgap of the Ga_2_O_3_ thin film increased from 4.69 eV to 4.90 eV, respectively. This increase in bandgap energy was attributed to the reduction of defects and impurities in the Ga_2_O_3_ thin film due to the annealing process. It is known that in Ga_2_O_3_ thin film, the donors are formed by oxygen vacancies and Ga interstitials, while acceptors are formed by Ga vacancies or a Ga-O vacancy pair [41,42,43]. As evident from the XPS O1s and Ga3d analysis depicted in Figure 2, oxygen vacancies generated during a high-temperature annealing process can introduce additional donor levels below the bandgap, resulting in a decrease in the effective bandgap. However, as depicted in Figure 1, during the high-temperature annealing process, when the amorphous Ga_2_O_3_ thin film transforms into β-Ga_2_O_3_ thin film, two-dimensional crystal defects, such as dislocations and grain boundaries, in addition to oxygen vacancies, are diminished. This reduction in crystal defects can further diminish the shallow levels around the bandgap. Consequently, the Ga_2_O_3_ thin film exhibits an energy bandgap closely aligned with the ideal energy bandgap of β-Ga_2_O_3_, approximately 4.9 eV [44].

**Figure 5:**
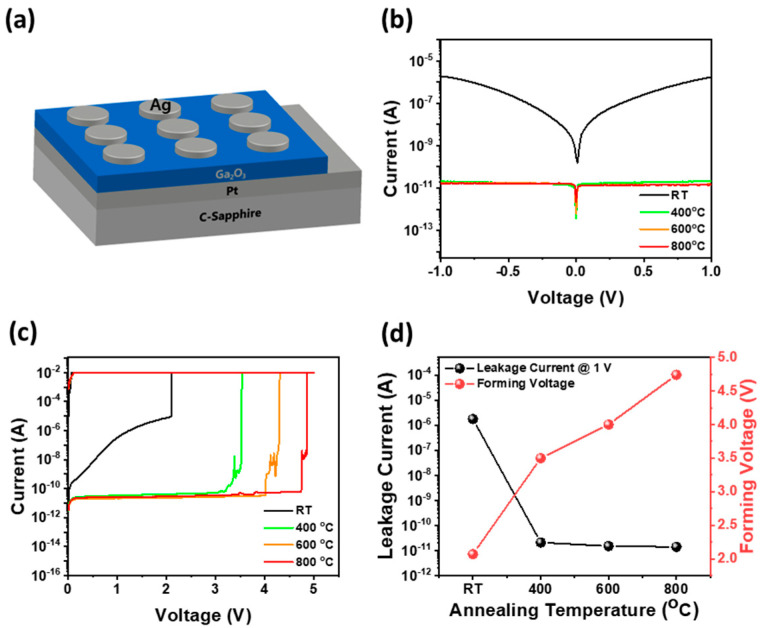
(**a**) Schematic of Ag/Ga_2_O_3_/Pt memristor structure grown on sapphire substrate. (**b**) Current (I)–Voltage (V) curves for Ag/Ga_2_O_3_/Pt memristor. (**c**) I–V curves of the forming process for HRS-LRS conversion of as-grown Ag/Ga_2_O_3_/Pt memristor and Ag/Ga_2_O_3_/Pt memristor annealed at different temperatures. (**d**) Leakage current at 1.0 V and forming voltage of the Ag/Ga_2_O_3_/Pt memristor as a function of annealing temperature.

Figure 5a shows a schematic of Ag/Ga_2_O_3_/Pt grown on c-plane sapphire memristor structure. Probe contacts were made to the top electrode, Ag, and the bottom electrode, Pt, respectively, to measure the electrical and memristive characteristics. Figure 5b shows the current (I)–voltage (V) curves obtained under applied bias ranging from −0.1 V to +0.1 V for a Ag/Ga_2_O_3_/Pt memristor structure containing Ga_2_O_3_ films annealed at various temperatures. The memristor demonstrated a current of +1.7 μA at 1.0 V. However, following an annealing process at temperatures exceeding 400 °C, the current drastically decreased to only a few tens of pA at +1.0 V. The substantial difference in leakage current was attributed to the significant reduction in crystal defects as the initially as-grown Ga_2_O_3_ thin film underwent transformation from its amorphous form to β-Ga_2_O_3_ crystallization through the annealing process [45]. In addition, as shown in Figure 4b, although high leakage current was observed initially due to the increased electron mobility facilitated by numerous crystal defects in the amorphous form near the bandgap of as-grown Ga_2_O_3_ thin films, the annealing process above 400 °C is believed to have mitigated these crystal defects and enhanced insulating properties by promoting recrystallization from amorphous to β-Ga_2_O_3_ thin film. This transformation resulted in a lower operating current for the memristors with the Ag/Ga_2_O_3_/Pt structure.

Figure 5c illustrates the I–V curves depicting the forming process of Ag/Ga_2_O_3_/Pt memristors annealed at different temperatures. The forming process consisted of applying an applied voltage, increasing from 0 V to 5 V, followed by a decrease back down to 0 V. In memristors, the forming process is the initial operation required to form conductive filaments necessary for the transition from the HRS to the LRS [46]. An as-grown Ga_2_O_3_ memristor without the annealing process can transition from HRS to LRS at voltages as low as about 2.07 V. However, Ga_2_O_3_ memristors that have been annealed at temperature above 400 °C must undergo the forming process at voltages above 3.5 V. Figure 5d shows that as the annealing temperature of the as-grown Ga_2_O_3_ memristor increased from 400 °C to 800 °C, the forming voltage increased from 3.5 V to 4.74 V, and the leakage current gradually declined from 21 pA to 14 pA, respectively. This trend can be attributed to the crystallization of the amorphous thin film into Ga_2_O_3_ thin film as the thermal treatment progresses. The abundance of crystal defects in amorphous, as-grown Ga_2_O_3_ thin film involves relatively easy migration of electrons [47]. Therefore, the forming process involving the application of high voltage to form conductive filaments is not necessary. However, as the annealing temperature increases, the transformation of the amorphous Ga_2_O_3_ thin film into a β-Ga_2_O_3_ thin film with crystalline properties occurs, rendering it challenging for oxygen vacancies and Ag^+^ ions to migrate. Consequently, a forming process is required to create conductive filament channel through which current can flow at high voltages [48].

**Figure 6:**
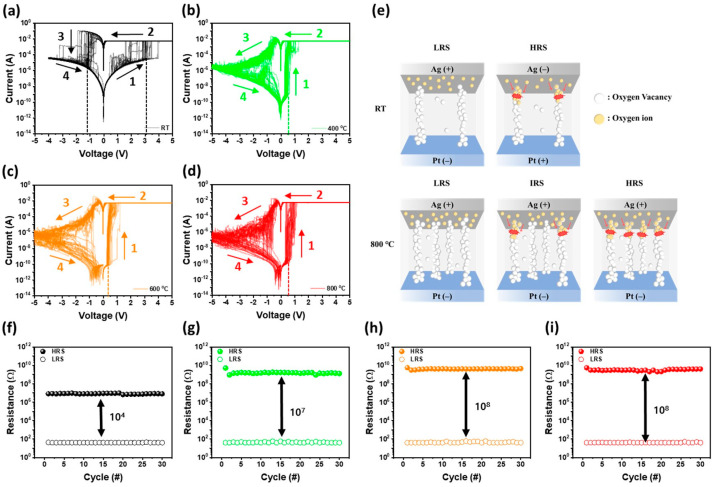
RS behaviors of I–V curves measured for 30 repetitions for (**a**) as-grown Ag/Ga_2_O_3_/Pt memristor and annealed Ag/Ga_2_O_3_/Pt memristors with different temperatures of (**b**) 400 °C, (**c**) 600 °C, and (**d**) 800 °C. (**e**) The schematic diagram of the formation of conductive filaments in the HRS and LRS of as-grown Ag/Ga_2_O_3_/Pt memristors, and in the HRS, intermediate resistive state (IRS), and LRS of 800 °C-annealed Ag/Ga_2_O_3_/Pt memristors. On/off ratio between HRS and LRS for (**f**) as-grown Ag/Ga_2_O_3_/Pt memristor and (**g**) 400 °C-, (**h**) 600 °C-, (**i**) 800 °C-annealed Ag/Ga_2_O_3_/Pt memristors as a function of the measurement cycle.

Figure 6a–d shows the resistive switching (RS) behaviors of bipolar memristors with an Ag/Ga_2_O_3_/Pt structure composed of as-grown Ga_2_O_3_, 400 °C-, 600 °C-, and 800 °C-annealed Ga_2_O_3_ thin films, respectively. The RS curves depict four bias steps: 0.0 V to 5.0 V (step 1), 5.0 V to 0.0 V (step 2), 0.0 V to −5.0 V (step 3), and −5.0 V to 0.0 V (step 4). During the first step (0.0 V to 5.0 V), a set process occurred, and the transition from HRS to LRS occurred due to the formation of conductive filaments. As depicted in Figure 6a, the as-grown Ga_2_O_3_ memristor exhibited a set voltage from the HRS to the LRS at an average of 3.0 V, which is comparable to the forming voltage shown in Figure 5c. As a result, it is believed that as-grown Ag/Ga_2_O_3_/Pt memristor is a free-forming device. However, the annealed Ga_2_O_3_ memristor displayed a set process at less than 1.0 V, significantly lower than the forming voltage after the forming process. The forming voltage of Ga_2_O_3_ memristors annealed at 400 °C or higher increased with the rise in annealing temperature. However, after the forming process, the set voltage remained almost consistent regardless of the annealing temperature. In Figure 6a, during the reset process from 0.0 V to −5.0 V, the as-grown Ga_2_O_3_ memristor exhibited a sharp transition to the HRS at an average of −1.1 V. This reset process caused the as-grown Ga_2_O_3_ memristor to transition from LRS to HRS. However, the Ga_2_O_3_ memristor subjected to thermal treatment at temperature above 400 °C, regardless of the annealing temperature, demonstrated a reset process starting at −0.4 V. This reset process gradually switched to the HRS until −5.0 V was reached. Subsequently, upon applying an injection voltage from −5.0 V to 0.0 V, the HRSs are observed. Following this, by applying forward and reverse bias, the previously observed set and reset processes were repeatedly demonstrated, showing the RS behaviors. The annealed Ga_2_O_3_ memristors demonstrated lower set voltages compared to those of the as-grown Ga_2_O_3_ memristor. As indicated by the XPS results of Figure 2, this is presumed to be attributed to the increase in oxygen vacancies during the annealing process, which enhances the migration of Ag^+^ ions to form readily conductive filaments, thereby resulting in lower set voltages. In general, in metal/Ga_2_O_3_/metal memristors, the formation mechanism of conductive filaments is attributed to the migration of oxygen vacancies and metal ions [49]. Therefore, it is believed that the high-quality annealed-Ga_2_O_3_ memristors with a higher concentration of oxygen vacancies facilitate the formation of additional conductive filaments once the initial conductive filaments are formed through the forming process, ultimately leading to lower set voltages [50]. In particular, as-grown Ga_2_O_3_ memristors exhibit sharp set and reset processes, while annealed Ga_2_O_3_ memristors exhibit a lower set voltage and a reset process with gradually increasing resistance. This is speculated to be due to the fact that as-grown Ga_2_O_3_ memristors are formed with one or fewer conductive filament, while annealed Ga_2_O_3_ memristors are formed with multiple conductive filaments. The sharp set and reset processes observed in as-grown Ga_2_O_3_ memristor are believed to stem from the highly radical transition between the HRS and LRS in the amorphous Ga_2_O_3_ thin film. Only a very small number of conductive filaments were formed and broken at the weakest points by Ag^+^ ions, rather than oxygen vacancies, depending on the applied voltage. In contrast, as shown in Figure 1 and Figure 2, the annealed Ga_2_O_3_ thin films experienced a slight increase in oxygen vacancies [51], but the crystalline defects decreased as the crystallization processed from amorphous to β-Ga_2_O_3_, resulting in the formation of an excellent thin film. Consequently, it became challenging for initial conductive filaments to form, leading to a high forming voltage, as shown in Figure 5d. However, there remains the possibility that multiple conductive filaments can be formed by crystal defects such as grain boundaries crystallized from β-Ga_2_O_3_ when high voltage is applied. Therefore, it is speculated that the set process occurs radically at low voltage due to the increase in oxygen vacancies and the generation of multiple conductive filaments by Ag^+^ ions during the annealing process. Conversely, under reverse bias, a gradual reset process occurs as conductive filaments break due to the migration of Ag^+^ ions, and the thermal effect of high voltage application gradually takes effect. This gradual reset process offers greater controllability compared to a rapid reset, making memristors particularly suitable for analog memory applications, such as multi-bit operation, with excellent retention and durability [52]. Figure 6f–i shows the operating current of the HRS and LRS at 0.5 V for memristors with a Ag/Ga_2_O_3_/Pt structure composed of as-grown Ga_2_O_3_ and annealed Ga_2_O_3_ at different temperatures, with 30 measurements of resistive switching ranging from −0.5 V to + 5.0 V. The annealed Ga_2_O_3_ memristors exhibited larger HRS/LRS ratios compared to the as-grown Ga_2_O_3_ memristor due to lower leakage current in the HRS state. It is believed that the HRS/LRS ratio increases with rising annealing temperature because the as-grown Ga_2_O_3_ thin films crystallize from amorphous to β-Ga_2_O_3_ thin film through a high-temperature annealing process, as shown in Figure 1. This process slightly increases point defects, such as oxygen vacancies, as shown in Figure 2a, but decreases overall crystal defects, resulting in a higher HRS/LRS ratio at higher annealing temperatures [53]. The large HRS/LRS ratio and low set voltage observed in the Ag/Ga_2_O_3_/Pt memristor, resulting from the high-temperature annealing process for Ga_2_O_3_, indicate a clear distinction between the on and off states of the memristor and lower power consumption.

**Figure 7:**
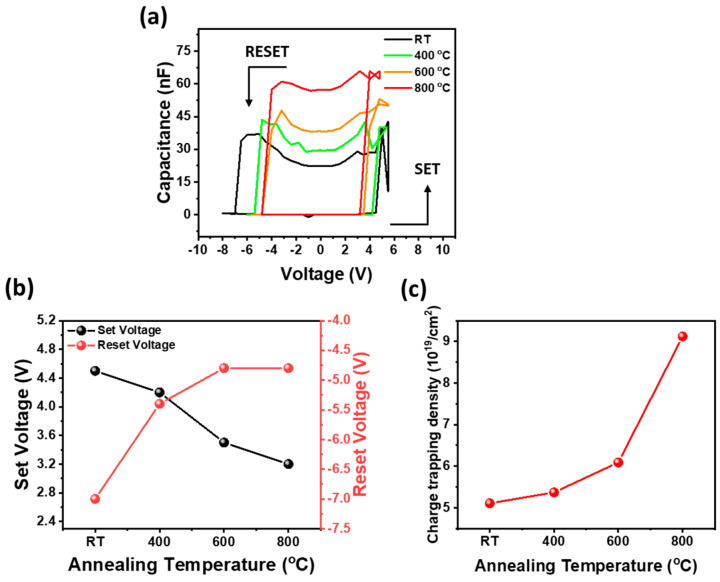
(**a**) C-V hysteresis curves representing the capacitive switching behavior of Ag/Ga_2_O_3_/Pt memristors heated at different temperatures into membranes, (**b**) set and reset voltage variations of Ag/Ga_2_O_3_/Pt membranes as a function of annealing temperature, and (**c**) charge trapping density of Ag/Ga_2_O_3_/Pt membranes as a function of different annealing temperatures.

Figure 7a shows the C-V curves depicting the capacitive switching behavior of the Ag/Ga_2_O_3_/Pt memristor functioning as a memcapacitor with applied voltage across the set and reset processes, where the set and reset processes represent the low capacitance state to high capacitance state and the high capacitance state to low capacitance state. Initially, the as-grown Ga_2_O_3_ memcapacitor exhibited a set voltage of 4.5 V and a reset voltage of −7.23 V. However, as the annealing temperature progressed beyond 400 °C, both the set voltage and reset voltage decreased. In particular, at 800 °C, the set voltage and reset voltage were measured to be 3.2 V and −4.8 V, respectively. Furthermore, it was observed that the maximum capacitance of the as-grown Ga_2_O_3_ memcapacitor significantly increased from 37 nF to 66 nF for the 800 °C-annealed Ga_2_O_3_ memcapacitor following the set process. This indicates that the as-grown amorphous Ga_2_O_3_ thin film can be positively affected, in terms of capacitance, by a high-temperature annealing process, thereby improving its charge storage capacity, and improving its performance as a memcapacitor. The observation that the maximum capacitance increases with the effect of the annealing process indicates that the electrical behavior and performance of the Ga_2_O_3_-based memristors and memcapacitor, which exhibit this capacitive switching behavior, are significantly influenced by the thermal treatment effect. In addition, to calculate the density of trapped charges (*N_charge_*) of memristors that can be used as memcapacitors in Ag/Ga_2_O_3_/Pt structures with Ga_2_O_3_ annealed at different annealing temperatures, the following equation was used:

$$N_{charge}=\frac{∆V\;\cdot \;C_{LRS}}{qA\;}$$

where Δ*V* is the difference between the set voltage and reset voltage, and *C_LRS_* is the capacitance in the LRS, *q* is the electronic charge (1.602 × 10^−19^ C), and *A* is the area of the device [54]. The charge capture density provides information about the density of charges trapped in the Ga_2_O_3_ thin film present between the Ag and Pt electrodes, which is directly related to the charge storage capacity of the Ag/Ga_2_O_3_/Pt memcapacitor that can also be used to form a memristor. The as-grown Ga_2_O_3_ memcapacitor exhibited a ΔV value of 11.5 V, but as the annealing temperature increased from 400 °C to 800 °C, it decreased from 9.6 V to 8.0 V, respectively. The *C_LRS_* value of the as-grown Ga_2_O_3_ memcapacitor was 22.4 nF. However, it increased significantly from 29.4 nF to 56.8 nF as the annealing temperature increased from 400 °C to 800 °C. As a result, as shown in Figure 7c, the trapped charge density of the as-grown Ga_2_O_3_ memcapacitor was 5.1 × 10^19^ cm^−2^, but with the increase in annealing temperature, the trapped charge density increased continuously, reaching 9.1 × 10^19^ cm^−2^ at 800 °C annealing temperature. This increase in the *N_charge_* value was attributed to the migration of Ag^+^ ions through the defect site, such as oxygen vacancies, and their trapping in high−purity Ga_2_O_3_ [55]. Therefore, it has a higher capacitance value because of the Ag^+^ ion trap generated at the defective site, which affects the performance improvement of the memristor and capacitor. Based on these results, it is speculated that higher annealing temperatures increase the *N_charge_* and the density of trapped charges, resulting in better charge storage and improved device performance.

## 4. Conclusions

This study demonstrates significant improvements in the thin film characteristics and the memristor through the annealing process of Ga_2_O_3_ thin films. As a result of XRD measurements, the phase transformation from an annealing-processed Ga_2_O_3_ thin film to β-Ga_2_O_3_ was confirmed. It was also confirmed, through absorbance analysis, that the amorphous Ga_2_O_3_ was changed to β-Ga_2_O_3_. Through XPS analysis, it was found that the oxygen vacancy density was higher in the annealed Ga_2_O_3_ thin film. Through the above two results, the leakage current rapidly decreased through recrystallization in the annealing Ga_2_O_3_ device, and the formation of a conductive film became easy due to the increase in oxygen vacancies, confirming a decrease in set voltage. It is crucial for conductive filament formation and the charge transport path in memristors. A Ga_2_O_3_ thin film with abundant oxygen vacancies exhibits decreased set voltage and increased capacitance in LRS, attributed to additional carriers such as oxygen vacancies and Ag^+^ ions. This indicates easy capacitance control, depending on channel presence. Based on these results, we suggest that annealing temperatures up to 800 °C improve the performance of Ga_2_O_3_ memristors. The thermal treatment at this temperature enhances the crystalline quality of the Ga_2_O_3_ thin films, reducing defects that typically hinder the formation of stable high-resistance and low-resistance states. Consequently, the memristor’s ability to switch between these states is improved, thereby enhancing its performance.

## Data Availability

The raw data supporting the conclusions of this article will be made available by the authors on request.
